# A Multi-Omic Systems-Based Approach Reveals Metabolic Markers of Bacterial Vaginosis and Insight into the Disease

**DOI:** 10.1371/journal.pone.0056111

**Published:** 2013-02-06

**Authors:** Carl J. Yeoman, Susan M. Thomas, Margret E. Berg Miller, Alexander V. Ulanov, Manolito Torralba, Sarah Lucas, Marcus Gillis, Melissa Cregger, Andres Gomez, Mengfei Ho, Steven R. Leigh, Rebecca Stumpf, Douglas J. Creedon, Michael A. Smith, Jon S. Weisbaum, Karen E. Nelson, Brenda A. Wilson, Bryan A. White

**Affiliations:** 1 Department of Animal and Range Sciences, Montana State University, Bozeman, Montana, United States of America; 2 The Institute for Genomic Biology, University of Illinois, Urbana, Illinois, United States of America; 3 Biotechnology Center, University of Illinois, Urbana, Illinois, United States of America; 4 Department of Microbiology, University of Illinois, Urbana, Illinois, United States of America; 5 Department of Anthropology, University of Illinois, Urbana, Illinois, United States of America; 6 Department of Animal Sciences, University of Illinois, Urbana, Illinois, United States of America; 7 J. Craig Venter Institute, Maryland Campus, Rockville, Maryland, United States of America; 8 The Department of Obstetrics and Gynecology, The Mayo Clinic, Rochester, Minnesota, United States of America; 9 Department of Obstetrics and Gynecology, Christie Clinic, Urbana, Illinois, United States of America; 10 Department of Obstetrics and Gynecology, Carle Clinic, Urbana, Illinois, United States of America; Columbia University, United States of America

## Abstract

**Background:**

Bacterial vaginosis (BV) is the most common vaginal disorder of reproductive-age women. Yet the cause of BV has not been established. To uncover key determinants of BV, we employed a multi-omic, systems-biology approach, including both deep 16S rRNA gene-based sequencing and metabolomics of lavage samples from 36 women. These women varied demographically, behaviorally, and in terms of health status and symptoms.

**Principal Findings:**

16S rRNA gene-based community composition profiles reflected Nugent scores, but not Amsel criteria. In contrast, metabolomic profiles were markedly more concordant with Amsel criteria. Metabolomic profiles revealed two distinct symptomatic BV types (SBVI and SBVII) with similar characteristics that indicated disruption of epithelial integrity, but each type was correlated to the presence of different microbial taxa and metabolites, as well as to different host behaviors. The characteristic odor associated with BV was linked to increases in putrescine and cadaverine, which were both linked to *Dialister* spp. Additional correlations were seen with the presence of discharge, 2-methyl-2-hydroxybutanoic acid, and *Mobiluncus* spp., and with pain, diethylene glycol and *Gardnerella* spp.

**Conclusions:**

The results not only provide useful diagnostic biomarkers, but also may ultimately provide much needed insight into the determinants of BV.

## Introduction

Mechanistic understandings of a number of diseases that are believed to have microbial axes have escaped conventional biological analyses [1]–[3]. The application of integrative systems biology approaches exploiting multiple data sources holds much promise in developing a fuller understanding of these diseases [4], [5].

Bacterial vaginosis (BV) is the most common vaginal disorder in reproductive age women. BV is characterized by a creamy grey discharge containing clue cells that typically has a pH>4.5 and an amine or “fishy” odor [6], [7]. BV is discomforting to women, and increases the risks of infertility, pre-term birth and the acquisition of sexually transmitted infections including HIV [8] making it of particular clinical interest. The underlying mechanism that causes BV remains unknown.

The vaginal microbiomes of healthy reproductive age women are often, but not always, dominated by lactic acid-producing *Lactobacillus* species [8], [9]. Women with BV commonly exhibit reductions in lactobacilli. *Gardnerella vaginalis* (formerly *Haemophilus vaginalis* or *Corynebacterium vaginale*) was first proposed as a bacterial cause of BV after it was found to be almost universally cultured from BV-diagnosed women [10]. Gram-stain-based methods described by Nugent [6] and Spiegel [7] have been used to delineate BV from other vaginal disorders or infections (vaginal candidiasis/trichomoniasis, gonorrhoeae, chlamydia). These methods apply a numbered ranking system from 1–10, where an increase reflects reductions in the relative proportions of *Lactobacillus* morphotypes and increases in *Gardnerella* and other Gram-variable rod shaped morphotypes. The initial method of Spiegel [7] was evaluated by Nugent [6] to be only slightly better than chance in predicting BV, while Nugent and colleagues reported an almost 20% false positive rate in describing an improved approach. Recent studies suggest just 16%–37% of women with a Nugent score indicative of BV actually show symptoms of the disease [11], [12]. A likely confounding factor of these approaches is that reductions in lactobacilli are also occasionally seen in asymptomatic individuals [9], [13] and *G. vaginalis* is commonly found in the vaginal tract, regardless of disease symptoms [9], [13], [14]–[16]. A more definitive diagnosis is based on the Amsel criteria [17], which diagnoses BV based on the presence of three of the four symptoms (discharge, odor, pH>4.5 or clue cells). Because of the limitations associated with the Nugent method, Amsel criteria are more commonly employed in a clinical setting and CDC guidelines preclude treatment without reported or observed symptomology. Despite this, Nugent score continues to be used in research as a diagnostic tool and this may have confounded our current understanding of BV.

Prior to the development of Gram stain-based methods, Spiegel and colleagues [18] favored volatile fatty acid (VFA) profiles of women exhibiting symptoms of BV and asymptomatic women, noting a strong correlation between the succinate to lactate ratio and disease state. However at the time, the equipment necessary to evaluate VFAs was not commonly available in many clinical laboratories [18].

Here we investigated the potential for modern biological techniques, including 16S rRNA gene-directed evaluations of community composition, and metabolomic profiles to adequately distinguish patients with BV from those without BV. Finally, we combined 16S rRNA gene and metabolite profiles with the patients' metadata and used a network-based approach to discern potential, but previously unrecognized, determinants of BV, leading to new and very testable hypotheses.

## Results

### 16S rRNA gene profiles

We began our multi-omic approach by performing deep sequencing using 454 FLX-titanium technology of the V1-V3 region of 16S rRNA genes amplified from vaginal lavage samples collected from 36 pre-menopausal women of reproductive age. The women sampled included five Amsel-positive women with a high Nugent score (≥7) and three Amsel-positive women with a low (<3; n = 2) or moderate (4–6; n = 1) Nugent score. The 28 Amsel-negative women also included low (n = 17), moderate (n = 7) and high (n = 4) Nugent scoring representatives. These women represented a broad range of self-reported age (20–45), ethnicity (African American, Asian American, and Caucasian), sexual behaviors, and hygiene practices (Table 1).

**Table 1:** Sample Descriptions.

Sample ID	BV Symptoms	Metabotype	Nugent Score	Biotype^1^	Ethnicity	Age	Marital Status	Last Period	Tampon Use	Pregnancies	Promiscuity	New Partner	Days Since Intercourse	Vaginal Sex Freq.	Oral Sex Freq.	Anal Sex Freq.	Condom Use	Bathing Freq.	Last Douching
401		II	0	III	C	29	M	7	Y	0	1	N	1	3–4	0–2	0	L	0–2	
402		II	0	II	C	24	S	24	Y	0	1	N	1	5–6	3–8	0	N	0–2	
403		II	6	IV	S	20	S	16	Y	0	2	Y	0	7+	12+	3–8	M	0–2	90
404		II	0	I	C	25	M	19	Y	0	1	N	7	0–2	3–8	0	N	0–2	
405		I	0	I	C	nr	S	nr	Y	0	0	N	nr	0	0	0	nr	0–2	
406		I	0	I	C	25	M	17	Y	0	1	N	2	0–2	9–12	0	nr	0–2	
408	I	I	0	I	C	nr	M	9	Y	1	1	N	45	0–2	0–2	0–2	N	0–2	
409		II	9	IV	C	22	S	30	Y	0	4+	Y	3	0–2	3–8	0–2	L	0–2	
412	DPICH	II	6	IV	A	26	S	15	N	2	1	N	10	7+	0	0	M	5–6	7
413	DPICH	II	1	I	C	nr	S	15	N	3	1	N	10	0–2	0–2	0–2	N	0–2	
416	DCH	II	3	III	C	22	S	20	Y	0	1	N	3	3–4	3–8	0	N	0–2	6
417	DOCH	II	7	IV^2^	A	30	S	13	N	0	1	N	25	0–2	0	0	M	0–2	180
418		I	1	I	C	23	S	7	Y	0	1	N	120	0–2	3–8	0	M	0–2	
423		I	1	I	C	24	M	9	Y	0	1	N	2	0–2	0	0	N	0–2	
424		I	0	I	C	36	S	22	N	0	2	Y	2	0–2	0–2	0	A	0–2	
425		I	0	I	C	33	M	30	N	0	1	N	2	3–4	0–2	0	L	3–4	
426		I	1	II	C	35	M	14	Y	4	1	N	1	3–4	3–8	0	N	0–2	
427		I	2	I	C	30	M	16	N	0	1	N	nr	0–2	0–2	0	A	0–2	
428		I	4	I	C	27	S	18	Y	0	1	N	3	0–2	0	0	A	0–2	
429		I	3	II	S	45	M	2	Y	4	1	N	7	0–2	0	0	N	7+	
431		I	7	II	C	24	S	30	Y	0	1	N	2	7+	3–8	3–8	N	0–2	
432		I	1	II	C	38	M	19	N	2	1	N	5	3–4	0	0	N	0–2	
433		I	6	II	C	30	S	25	Y	0	2	Y	45	0–2	0–2	0	A	7+	
434	DOCH	I	8	IV	C	46	S	16	N	1	1	N	4	3–4	0–2	0	N	3–4	14
435		I	5	V	A	37	M	27	Y	2	1	N	1	3–4	3–8	0	N	0–2	
436		I	0	I	C	28	S	17	Y	0	1	N	25	3–4	0–2	0–2	N	0–2	
437		I	7	V	C	37	M	19	Y	0	1	N	0	0–2	3–8	0	A	0–2	
438		I	3	I	C	29	M	24	Y	0	1	N	25	0–2	0	0	N	0–2	
439		I	10	IV	C	41	M	18	N	5	1	N	1	nr	0–2	0	N	0–2	
440		I	3	II	S	28	S	16	Y	0	1	N	5	0–2	0	0	N	0–2	
441		I	5	III	C	32	M	17	Y	3	1	N	1	0–2	3–8	0–2	N	0–2	
442		I	4	II	C	33	M	7	N	0	1	N	10	0–2	0	0	N	0–2	
443	DOPCH	I	8	III	C	25	S	22	Y	0	0	N	nr	0–2	0	0	L	7+	
CC001	DCH	II	7	IV	A	38	S	8	Y	6	1	N	1	0–2	0–2	0	N	0–2	
CC002		I	5	III	C	25	S	25	Y	2	1	N	4	0–2	0–2	0	N	0–2	
CC003	DOCH	I	9	II	A	41	S	17	Y	3	1	N	1	3–4	3–8	0–2	N	0–2	2

BV Symptoms: (D)ischarge, (O)dor, (P)ain, (I)tching, p(H), (C)lue cells; Ethnicity: (A)frican American, A(S)ian American, (C)aucasian; Marital status: (M)arried, (S)ingle/Divorced; Tampon use/New partner (in past 6 months): (Y)es, (N)o; Oral and Anal sex frequency is denoted as times monthly, Vaginal sex and Bathing frequency is weekly; Condom use: (N)o, (L)ess than half of the time, (M)ore than half of the time, (A)lways.

nr Not reported; ^1^Biotype as per Ravel *et al.*, 2011; ^2^Near equal numbers of *L. gasserii* were detected.

**Figure 1:**
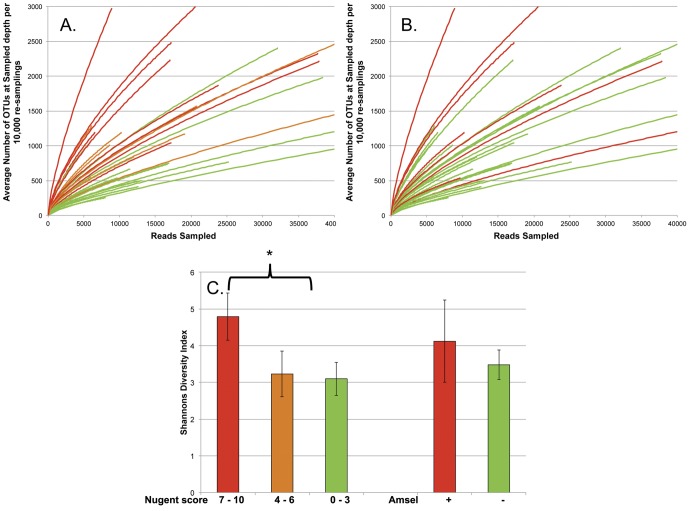
Richness and Diversity of Each Sample. Rarefaction curves showing the richness of microbiomes for all samples colored by Nugent score (A; green = 0–3, orange = 4–6, red = 7–10) or Amsel classification (B; red = positive, green = negative) are presented along with Shannon diversity indexes (C), with samples grouped by Nugent score or Amsel criteria and colored as in rarefaction curves.

Richness (average species observed per 5000 reads (SOBs_5K_): high Nugent score median  = 920.5 vs. low-moderate Nugent score median = 375.1; Fig. 1A) and diversity analyses using both Shannon's (high Nugent score median = 4.79 vs. low-moderate Nugent score median = 3.10; Fig. 1C) and Simpson's (high Nugent score median = 0.029 vs. low-moderate Nugent score median = 0.18; data not shown) diversity indexes indicated a significant increase in diversity (*p*<0.0003) and richness (*p* = 0.01) in women with a Nugent score≥7 (Fig. 1; Table 1). The same analyses (symptomatic BV median = 441.3 vs asymptomatic BV median = 689.1, SOBs_5K_; symptomatic BV median = 4.12 vs asymptomatic BV median = 3.48, Shannon's index; symptomatic BV median = 0.047 vs asymptomatic BV median = 0.059, Simpson's index) also indicated a general, though not significant increase in richness (p>0.2) and diversity (p = 0.1) in women displaying symptoms of BV (Fig. 1; Table 1).

**Figure 2:**
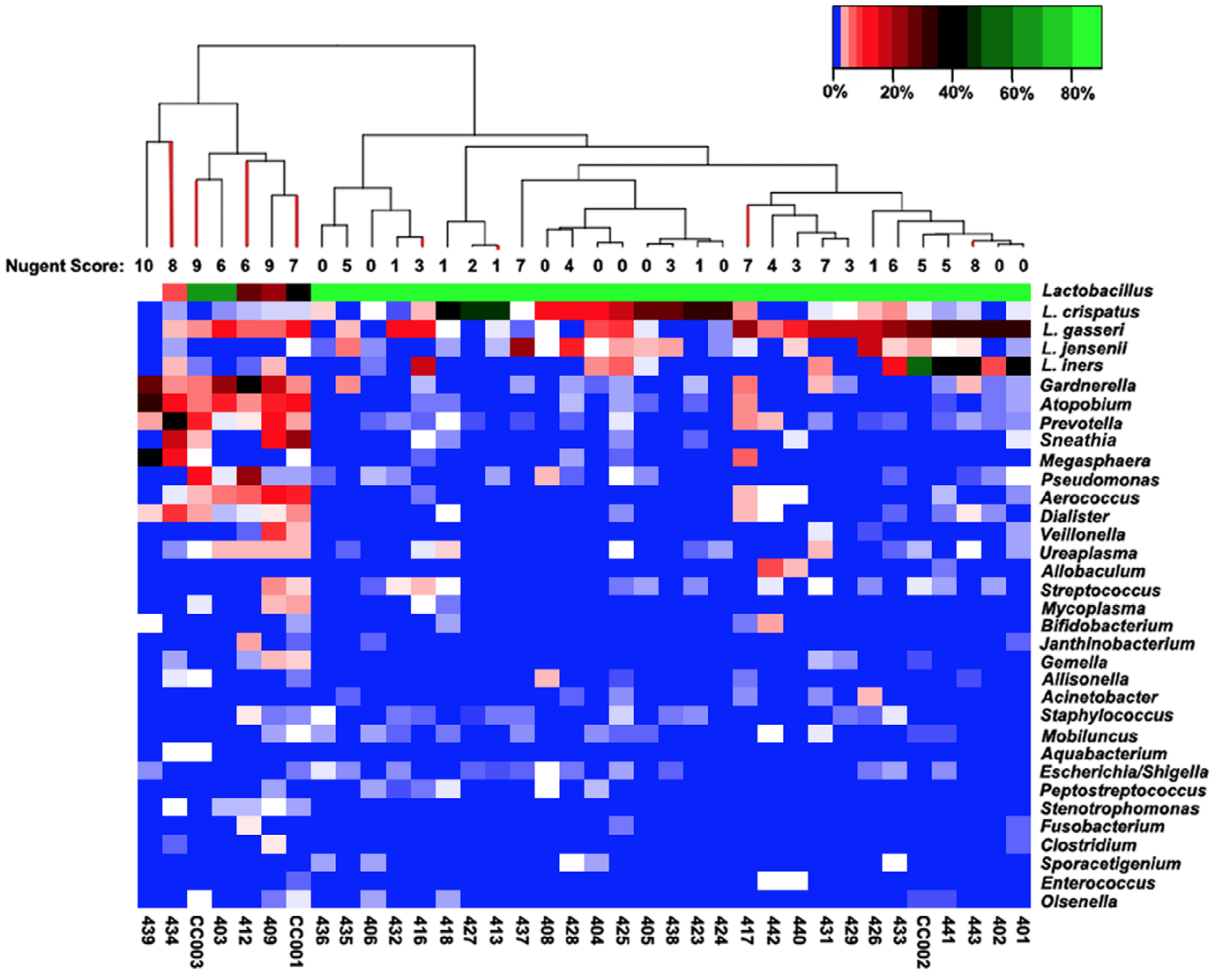
Heatmap of Taxonomic Enrichment by Sample. Shown is a heatmap of the relative enrichment of the most abundant thirty microbial genera across the entire sample set and the relationships among samples. Highly abundant genera tend toward bright green, while less abundant genera tend toward blue, as shown in the key. Dendrograms show the relationship among samples. Red bars in the dendrogram show the relationship of samples with symptomatic BV. Nugent scores are presented beneath the dendrogram.

We examined the genus-level composition of each sample (Fig. 2 and Table S1). 16S rRNA gene reads assigned to lactobacilli (p>0.7) were found in all samples ranging in proportions from 0.5% to >99.9%. We observed a marginal decrease in the maximum, median and average proportion of lactobacilli 16S rRNA gene reads in samples from women determined by the Amsel criteria [17] as having BV compared to samples from asymptomatic women, but it was not significant (p = 0.97). Equally, no significant differences were observed in the proportion of lactobacilli 16S rRNA gene reads among samples determined to have high, moderate or low Nugent scores (p>0.55) [6].

16S rRNA gene reads assigned to the genus *Gardnerella* were found in samples from 21 of the 36 subjects with proportions of *Gardnerella* ranging from 0.000006%–38% (median 0.74%) of reads. *Gardnerella* 16S rRNA reads were more common in women assessed by the Amsel criteria to have BV (88%) than others (50%) and in women found to have a high Nugent score (100%) compared to those with moderate (62.5%) or low (37%) Nugent scores. *Gardnerella* were significantly more abundant in samples presenting with high Nugent scores relative to those with low Nugent scores (p = 0.05), but were not significantly different among samples assessed by Amsel criteria [17] to have BV or not (p = 0.4).

*Dialister* was the only other genus-level taxon found to be significantly more abundant in samples with high Nugent scores relative to those with low or moderate Nugent scores (p = 0.05). However, again the proportions of 16S rRNA reads assigned to this genus were not significantly different among samples assessed by Amsel criteria to have BV or not.

Ravel *et al.* [9] recently reported five distinct vaginal microbial biotypes, characterized by the dominance of *Lactobacillus crispatus* (I), *L. gasseri* (II), *L.* iners (III), *L. jensenii* (V), or an increased proportion of other strictly anaerobic bacteria (IV). No read within our data set was assigned by speciateIT (algorithm described in [9]) to *L. iners*, the dominant species of vaginal biotype III communities [9]. Consistent with this, a blast search using the 16S rRNA gene sequence of the *L. iners* type strain (CIP109878; Genbank accession HE573916) as the query and our dataset as a database yielded no match within 3% sequence ID. This was unexpected as *L. iners* had previously been detected in four of the eight samples from this dataset that were previously evaluated using full-length clone libraries [13]. We therefore used *L. iners*-specific primers to qualitatively test for *L. iners* among our samples. Using this approach *L. iners* was detected in 17 of the 36 samples (Fig. S1). We proceeded to quantitate *L. iners* 16S rRNA genes in these samples using qPCR, with the results presented in Table 2. Even when accounting for *L. iners* our total numbers of lactobacilli exceeded those collectively contributed from *L. crispatus, L. gasseri*, *L.* iners, and *L. jensenii*. This may indicate the common presence of other *Lactobacilli* species, or may simply reflect reads that could not be accurately classified to the species level.

**Table 2:** Quantitative PCR Enumeration of Lactobacillus inners by Sample.

Sample ID^1^	Copy number (bacteria/ml)	Standard Deviation	Standard Error
401	1.60E+08	5.08E+07	2.93E+07
402	2.75E+07	1.32E+07	7.61E+06
404	1.62E+07	1.60E+07	9.26E+06
405	6.78E+05	1.23E+05	7.09E+04
406	3.24E+04	6.42E+03	3.71E+03
409	5.14E+06	6.02E+05	3.48E+05
412	2.55E+04	4.42E+04	2.55E+04
416	7.64E+07	6.26E+06	3.62E+06
425	2.15E+07	1.30E+07	7.52E+06
426	7.72E+06	5.48E+06	3.16E+06
431	1.59E+07	2.08E+07	1.20E+07
433	3.79E+07	1.69E+07	9.78E+06
434	6.39E+06	5.72E+06	3.30E+06
441	1.80E+08	2.38E+07	1.37E+07
443	1.59E+08	1.44E+08	8.30E+07
cc002	2.08E+08	9.80E+07	5.66E+07
cc003	3.68E+04	1.57E+04	9.05E+03
			

1 Only samples found to contain *L. iners* are shown

We then evaluated the vaginal biotypes of our cohort using the same methodology as Ravel and colleagues [9] substituting in the qPCR data for *L. iners* as described in the methods. The majority of our samples were found to be either biotypes I (36%), II (25%) or IV (19%), biotypes III (14%) and V (6%) were also observed.

Specifically considering the relative representation of *Lactobacillus* species that define biotypes I, II, III and V, we found significant reductions in the proportion of *L. crispatus* among samples with high compared to low Nugent scores (p = 0.001).

**Figure 3:**
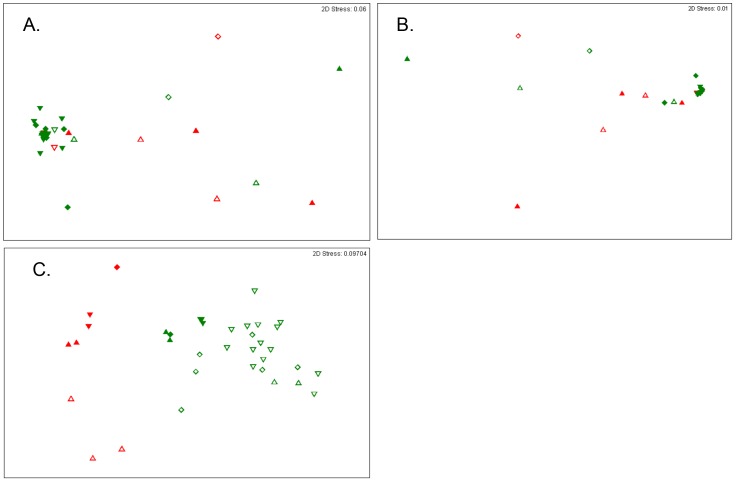
Nonmetric Multidimensional Scaling Analyses. Shown are nMDS plots of the 16S rRNA reads clustered by genus for all taxa (A), or for just *Gardnerella* and *Lactobacillus* (B), or metabolite profiles (C). Samples from patients determined by Amsel criteria to have BV are shown in red, while samples determined by Amsel to be healthy are shown in green. Samples with a high (7–10; up-pointing triangles), moderate (4–6; diamonds) and low (0–3; down-pointing triangles) are indicated. The two metabotypes are delineated by hollow (type I) and solid (type II) symbols.

We then applied non-metric multidimensional scaling (nMDS) to a Bray-Curtis dissimilarity matrix created from the genus-level taxonomic classifications normalized across the dataset (Fig. 3). Using this approach we found no clear separation among samples from women determined by Amsel criteria to have BV or those from other samples. However, we did observe some discrimination among women with high or low Nugent scores (Fig. 3A). Analysis of similarities (ANOSIM) supported this separation (ANOSIM R = 0.652, p = 0.0002). Focusing these approaches to the proportions of 16S rRNA reads determined to be from *Lactobacillus* or *Gardnerella* species improved the separation of low and high Nugent categories (ANOSIM R = 0.724, p = 0.0001; Fig. 3B), but failed to improve delineations based on Amsel criteria. In both analyses, samples with a moderate Nugent score were not significantly different from either low or high Nugent scored samples (R<0.34, p>0.8). Our ability to discriminate disease states did not improve using species-level (3% sequence similarity) Operational Taxonomic Units (OTUs; data not shown).

### Metabolomic characterization of BV

**Figure 4:**
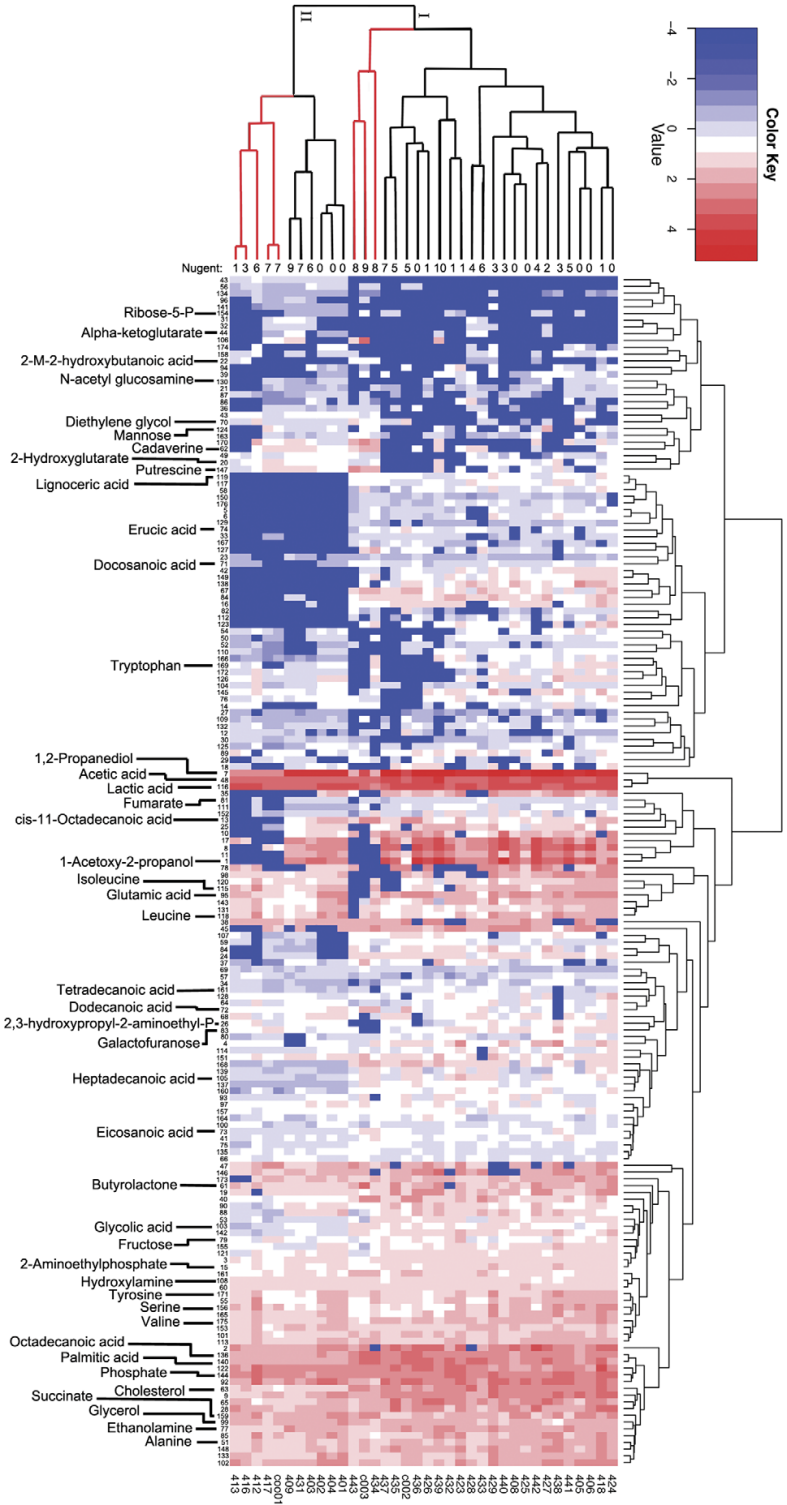
Heatmap of Metabolite Enrichment by Sample. Shown is a heatmap of the relative enrichment of each metabolite across the entire sample set and the relationships among samples and among metabolites. Highly abundant metabolites tend toward red, while less abundant metabolites tend toward blue. Dendrograms show the relationship among samples (top) and among metabolites (left). The two BV metabotypes observed (I and II) are labeled at their branching point. Red bars in the top dendrogram show the relationship of samples with symptomatic BV. Nugent scores are shown for each sample. Metabolite mentioned in-text are labeled, others are numbered as in Table S2.

**Figure 5:**
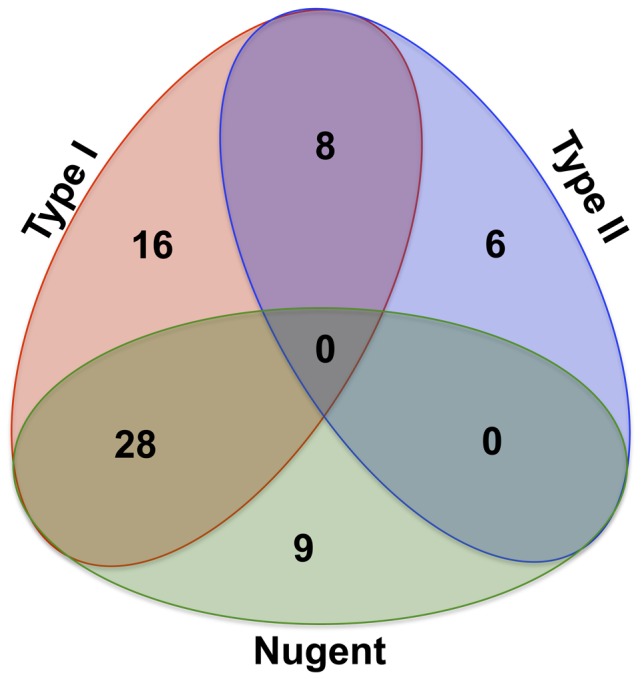
Venn Diagram of Discriminative Metabolites. Shown is a Venn diagram of the numbers of unique and shared metabolites that delineate high Nugent scoring (≥7), Type I symptomatic BV or Type II symptomatic BV from other samples.

Next we sought a metabolic description of BV. We subjected each of the samples to GC-MS analysis using both derivatized and underivatized lavage samples to examine the relative abundances of both volatile and non-volatile metabolites (Table S2). We identified 176 distinct metabolites across the 36 samples (Fig. 4). We used the resulting data to construct a Bray-Curtis dissimilarity matrix, which resulted in nMDS and ANOSIM analyses that had significant increases in discriminatory power (ANOSIM R>0.895, p<0.001; Fig. 3D). The vaginal metabolomes fell into two broad clusters (Fig. 4) distinguished by 48 metabolites, which we will refer to as metabotypes I and II (Table 3). Samples from women displaying symptoms of BV formed discrete subgroups within each metabotype (henceforth referred to as SBVI and SBVII). The two symptomatic BV-types were also evident in the nMDS analysis (Fig. 3D). Samples with a Nugent score≥7 fell into both metabotypes, capturing all SBVI samples, but less than half of the SBVII cases. We observed significant differences in the relative concentrations of a total of 67 metabolites among samples with high Nugent scores (n = 37), SBVI (n = 52), or SBVII (n = 14), and other samples (>3-fold and p <0.05; Fig. 5 and Table 4). SBVI and SBVII shared eight metabolites (Fig. 5). Nearly all significantly affected metabolites from samples with high Nugent scores and the majority of those affected metabolites in SBVI and SBVII, including those shared between the two symptomatic BV types, corresponded to decreases of the respective metabolite in the defined diseased state. The exceptions were putrescine, cadaverine, 2-methyl-2-hydroxybutanoic acid, hydroxylamine, glycolic acid, tetradecanoic acid (significantly affected in SBVI) and butyrolactone (significantly affected in SBVII), all of which were significantly increased in both symptomatic BV metabotypes. Eight metabolites were not detected in either SBVI or SBVII, but were found in all other samples (Table 4). This included the metabolites, 2,3-hydroxypropyl-2-aminoethyl phosphate (type I), cis-11-octadecanoic acid, and ribose-5-phosphate, whose relative concentrations were low, and varied to such an extent within the Amsel-negative samples that they were not detected as significant.

**Table 3:** Metabotype defining Metabolites.

Metabolite	Metabotype	Enrichment
1-Acetoxy-2-Propanol	I	1566.6
1-Hydroxy-2-Propanone	I	352.1
1-Methyl-beta-D-galactopyranoside	I	15.5
1,2-Propanediol	I	27196.1
1,2-Propanediol, 2-acetate	I	806.3
1,3-Dihydro isobenzofuran^D^	I	267.1
1,4-Dimethyldioxane	I	53.2
2-Aminoethylphosphate	I	6.2
2-Ethyl-4-methyl-1,3-Dioxolane	I	265.5
2-Hydroxy-gamma-butyrolactone	I	24.1
2-Methylbenzoic acid	I	13.5
2-Methylbenzoic acid	I	13.5
2-O-Glycerol-beta-D-galactopyranoside	I	15
2(5H)-Furanone^D^	I	66.7
3-Pyridinecarboxamide	I	62.5
9-Octadecenoic acid^D^	I	20.8
a-Tocopherolacetic acid	I	92.8
Acetate^D^	I	1934.2
Alanine	I	28.3
Arabitol	I	11.3
Butyrolactone	I	32.9
Cholesterol^D^	I	175.4
Cyclopentanol	I	136.6
Ethanolamine	I	23.4
Formate	I	19.7
Galactofuranose^D^	I	3.1
Galactopyranose^D^	I	15.4
Gluconic acid-1,5-lactone	I	4.8
Glucopyranose	I	6.9
Glycolic acid	I	6.3
Heptadecanoic acid^D^	I	6.2
Homoserine^D^	I	3.3
Octadecanoic acid^D^	I	290.1
Octadecanol^D^	I	3.2
Palmitic acid^D^	I	425.1
Pentadecanoic acid	I	10.9
Phosphate^D^	I	148.4
Propionate	I	37
Pyroglutamic acid	I	60.5
Pyruvate^D^	I	3
Sucrose^D^	I	4.6
Tetradecanoic acid^D^	I	12.8
Triethanolamine^D^	I	16.5
Tryptophan^D^	I	3.7
Urea^D^	I	21.1
Valine	I	30
		
Butylamine	II	5.9
Hydroxylamine	II	14.7

D Metabolite linked to douching in network.

**Table 4:** Significantly-affected Metabolites (Non-BV vs BV).

Metabolite	Metabolite Increased in	Median Enrichment		
		Nugent BV	SBVI	SBVII
1-Acetoxy-2-propanol	Non-BV^A^		1987.7**^2^	483.65**^2^
1-Hydroxy-2-propanone	Non-BV	228.6^4^	484^7^	
1-Methyl-β-D-galactopyranoside	Non-BV	10.6^28^		
1,2-Propanediol	Non-BV		51541.15^1^	23167.3^1^
1,2-Propanediol, 2-acetate	Non-BV		1069.5**^4^	
1,3-Dihydroisobenzofuran (Phthalan)	Non-BV	242.4^3^	305.55^8^	
1,3-Dipropylene glycol	Non-BV			8.65**^11^
1,4-Dimethyldioxane	Non-BV		171.6**^12^	116.7**^3^
2-aminoethylphosphate	Non-BV		10.4^41^	
2-Desoxy-pentos-3-ulose	Non-BV		6.7^47^	
2-Ethyl-4-methyl-1,3-dioxolane	Non-BV		545.25^5^	
2-Hydroxy-3-methyl-2-cyclopenten-1-one	Non-BV		4.4*^50^	
2-Hydroxy-γ-butyrolactone	Non-BV		43.15^28^	
2-O-Glycerol-β-D-galactopyranoside	Non-BV		22.9^35^	9.65^10^
2(5H)-Furanone	Non-BV	61.8^10^	96.8^15^	
3-Pyridinecarboxamide	Non-BV	61^11^	80.5^19^	
4-Hydroxybutanoate	Non-BV			3.15^14^
Acetate	Non-BV		1863.3^3^	
Alanine	Non-BV	27.1^22^		
Aspartic acid	Non-BV	51.8^12 = ^	46.8^25^	
Cholesterol	Non-BV	20.7^24^	43.3^27^	
Cyclopentanol	Non-BV		175.9^9^	
Dehydroascorbic acid	Non-BV	6.8^34^	6.2^48^	
Ethanolamine	Non-BV		94.2^16^	113.85^4^
Formate	Non-BV	38.1^17^	85.85^18^	
Fructose	Non-BV		3.7^51^	
Galactose	Non-BV	66.9^9^	86.2^17^	
Gluconic acid	Non-BV		18.6^36^	18.75^7^
Gluconic acid-1,5-lactone	Non-BV		9.45^43^	12.1^9^
Glucose	Non-BV	468.3^2^	498.4^6^	
Glutamate	Non-BV	163.2^6^	175.7^10^	
Glycerate	Non-BV			6.5^12^
Glycerol	Non-BV	38.6^16^	46.35**^26^	57.85^5^
Glycine	Non-BV	49.3^14^	30.9^33^	
Isoleucine	Non-BV	44.1^15^		
Lactate	Non-BV	1647.5^1^		
Leucine	Non-BV	67.5^8^	50.15^24^	
Lysine	Non-BV	21.5^23^	25.45^34^	
Malate	Non-BV	4.5^36^	9.9 ^42^	
Maltose	Non-BV		171.85^11^	
Methionine	Non-BV	12^27^		
Myo-inositol	Non-BV	9.5^29^		
N-Acetyl-lysine	Non-BV	14.3^25^	8.4^45^	
N-Acetylglutamate	Non-BV	51.8^12 = ^	35.35^31^	
Ornithine	Non-BV	7.7^33^	12.65^39^	
Phenylalanine	Non-BV	69.5^7^	60.6^22^	
Phosphate	Non-BV	167.1^5^	167.4^13^	
Proline	Non-BV		4.95^49^	
Propionate	Non-BV			36.55^6^
Pyroglutamate	Non-BV	35.2^18^	71.35^21^	
Pyrophosphate	Non-BV	4.3^37^		
Ribose	Non-BV	9.1^31^	14.2^38^	
Sedoheptulose	Non-BV	9.2^30^	8.7^44^	
Serine	Non-BV	32^21^	18.35^37^	
Sorbitol	Non-BV			4.2^13^
Threonine	Non-BV	35^19^	38.8^29^	
Tryptophan	Non-BV	6.1^35^		
Tyrosine	Non-BV	13.9^26^		
Urea	Non-BV		34.85^32^	
Valine	Non-BV	33.2^20^	38.75^30^	
2-Methyl-2-hydroxybutanoic acid	BV		3.3^52^	
Butyrolactone	BV			18.7^8^
Cadaverine	BV		128^14^	
Glycolic acid	BV		10.7^40^	
Hydroxylamine	BV		8.2^46^	
Putrescine	BV		73.2^20^	
Tetradecanoic acid	BV		55.35^23^	

A Non-BV by respective criteria ^B^ Metabolite was increased in Type II BV and Nugent-defined Non-BV ^1–59^ Represent rank order for each BV type * Metabolite not seen in BV types but seen in most or ** all non-BV types. Blank squares indicate either less than 2-fold enrichment or *p*-value>0.05 or both.

### Using networks to better understand BV

Exploiting all the collected experimental and demographic data, we applied a network approach to ascertain the linear parametric relationships among relative taxon or metabolite abundances; parametric patient metadata and Nugent scores; and their non-parametric relationships with patient demographics, symptoms and non-parametric metadata (Fig. S2 and Table S3). Using randomized variables, we determined that significance thresholds of ≥0.6 or negative correlations of ≤−0.4 for either Pearson's (parametric) or Spearman's (non-parametric) correlation coefficients were most informative of significant correlations (Fig. S3 and Fig. S4). A network constructed from these data revealed a complex web of interactions defining the BV state.

In the resulting network, Nugent score was inversely correlated with lactic acid (i.e. an increase in Nugent score = a decrease in lactic acid; Pearson's R = −0.68), but was only indirectly connected via glutamic acid (Spearman's R = −0.74) to symptomatic BV, as defined by Amsel criteria (Spearman's R = −0.58; Fig. S5). Nugent score was not correlated with any of the characteristic BV symptoms (discharge, odor, vaginal pain, itching), but was inversely correlated with *L. crispatus* (Pearson's R = −0.49) and Ravel's biotype I [9] (Spearman's R = −0.62). Both a high Nugent score (Spearman's R = −0.60) and BV as determined by Amsel criteria (Spearman's R = −0.41) were negatively correlated with sedoheptulose (Fig. S5A), which was inversely correlated with Ravel’s biotype IV [9] (Spearman’s R = −0.47) and the undertaking of anal sex (Spearman’s R = −0.51).

**Figure 6:**
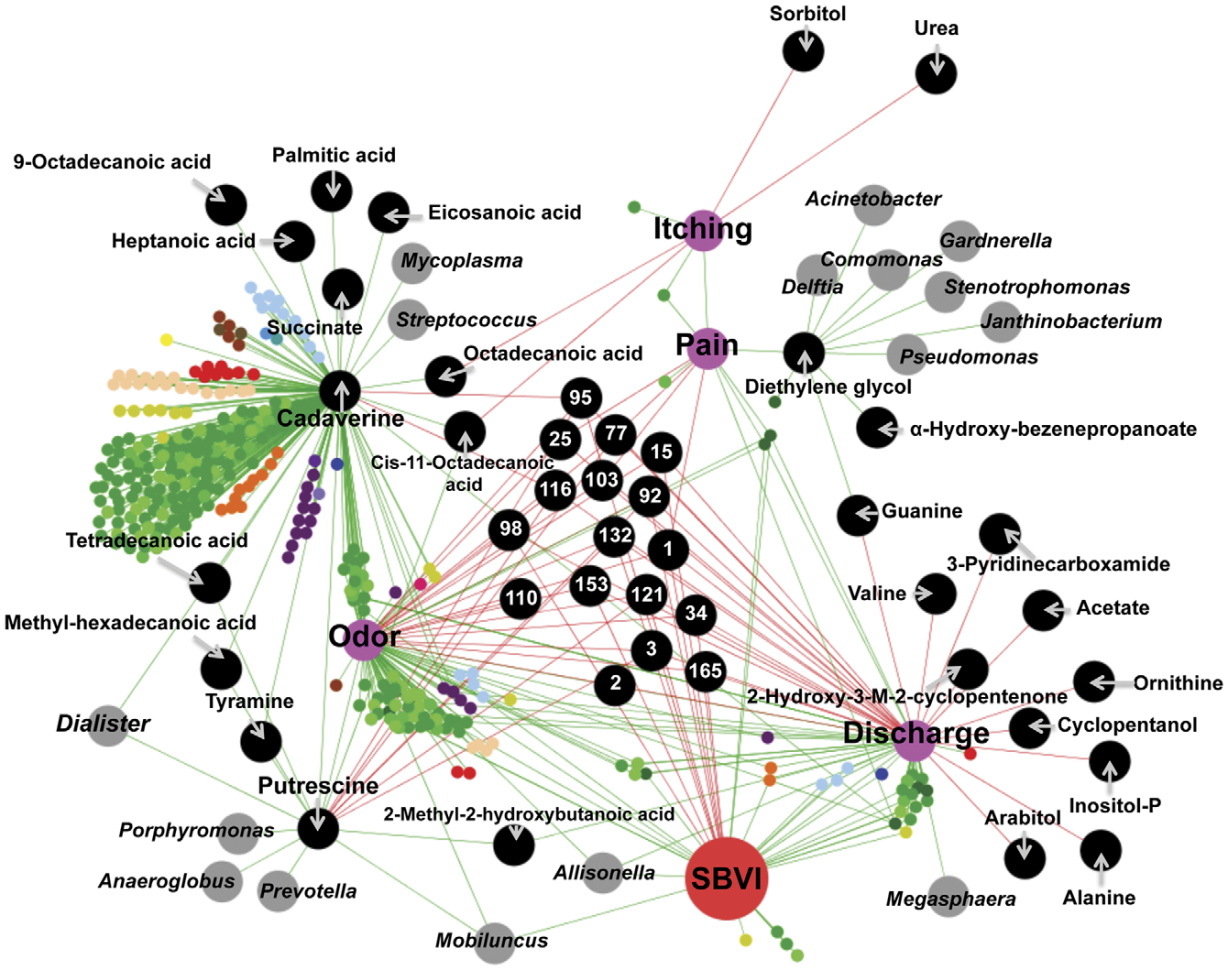
SBVI Sub-Network View of Linear Relationships among Variables. Pearson’s (between parametric data) and Spearman's (between non-parametric and either parametric or non-parametric data) correlations >0.6 (green) or <−0.4 (red) are shown as edges connecting patient metadata relating to demographics, hygiene and sexual behaviors and sexual practices, OTUs, microbial genera, metabolites and patient symptoms. Figures presented represent sub-networks of the complete network (Fig S2). Node identities are listed or described in the key for figure 7. The identities of numbered metabolites are listed in Table S2.

**Figure 7:**
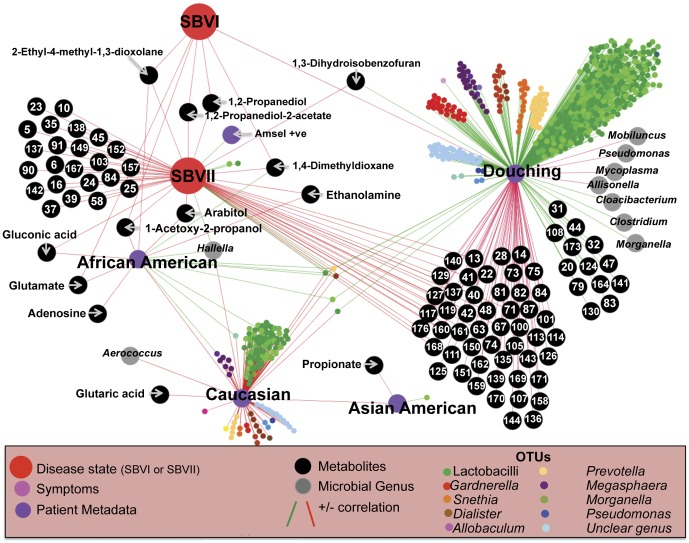
SBVII Sub-Network View of Linear Relationships among Variables. Pearson's (between parametric data) and Spearman's (between non-parametric and either parametric or non-parametric data) correlations >0.6 (green) or <−0.4 (red) are shown as edges connecting patient metadata relating to demographics, hygiene and sexual behaviors and sexual practices, OTUs, microbial genera, metabolites and patient symptoms. Figures presented represent sub-networks of the complete network (Fig S2). Node identities are listed or described in the key. The identities of numbered metabolites are listed in Table S2.

SBVI and SBVII formed direct links to numerous metabolites and certain microbial genera. SBVI was positively correlated to the genera *Mobiluncus* (Spearman’s R = 0.80) and *Allisonella* (Spearman’s R = 0.61), while negatively correlated with 15 distinct metabolites (Fig. 6). SBVII was positively correlated with *Hallella* (Spearman’s R = 0.60), while negatively correlated with 53 metabolites. Interestingly, African American women were indirectly connected to SBVII through *Hallella* (Spearman’s R = 0.6; Fig. 7). All but one BV-affected African American women were determined to be SBVII, although ethnicity was not the sole defining determinant, as two non-African American women were also SBVII.

Douching (parametrically determined as days since last douche in women who douche and contrasted to women who do not douche) and SBVII shared common negative connections to 21 different metabolites. However, as was observed for ethnicity, douching was not a universal determinant of SBVII. Douching appeared to have a highly perturbing effect on the vaginal environment. Douching correlated with changes in the abundance of 64 metabolites (Fig. 7), including increased levels of several sugars (e.g., Fructose, Mannose, and Galactofuranose), 2-hydroxyglutarate, α-ketoglutarate, and N-acetyl-glucosamine (a predominant component of mucin), and reductions in many fatty acids (e.g., Docosanoic acid, Dodecanoic acid, Eicosanoic acid, Erucic acid, Heptadecanoic acid, Lignoceric acid, Octadecanoic acid, Palmitic acid, Succinate and Fumarate), fatty acid derivatives and cholesterol. Douching was negatively correlated with *Mobiluncus* spp. (Pearson's R = −0.43), *Pseudomonas* spp. (Pearson's R = −0.47), *Mycoplasma* spp. (Pearson's R = −0.44) and *Allisonella* spp. (Pearson's R = −0.42) and was positively linked to increases in the genera *Cloacibacterium* (Pearson's R = 0.89), *Morganella* (Pearson's R = 0.89) and *Clostridium* (Pearson's R = 0.64).

Douching also did not appear to separate metabotypes I and II, affecting just nineteen of the 48 metabolites separating the two metabotypes (Table 3). Although not correlated with SBVI or SBVII, *Veillonella* (Spearman's R = −0.53), *Gardnerella* (Spearman’s R = −0.50), *Aerococcus* (Spearman’s R = −0.47), *Atopobium* (Spearman’s R = −0.45), and *Peptostreptococcus* (Spearman’s R = −0.41), along with Ravel’s biotype IV (Spearman’s R = −0.44), were all negatively correlated with metabotype I.

We found correlative links among symptoms, microbial genera, and metabolites (Fig. 6). The symptoms ‘itching’ and ‘vaginal pain’ were linked (Spearman’s R = 0.64) to each other, as well as to diethylene glycol (Spearman’s R>0.6) and several distinct metabolites. The symptoms ‘odor’ and ‘discharge’ were linked (Spearman’s R = 0.79) to each other, but were separate from ‘itching’ and ‘vaginal pain’ (Fig. 6). Odor was strongly associated with increased levels of putrescine (Spearman’s R = 0.84) and cadaverine (Spearman’s R = 0.82), and was linked to SBVI (Spearman’s R = 0.85), but not to SBVII (Spearman’s R = 0.11). Discharge was more strongly linked to the metabolite 2-methyl-2-hydroxybutanoic acid (Spearman’s R = 0.73), and was strongly linked to all samples described as BV by the Amsel criteria (Spearman’s R = 0.84).

Putrescine (Spearman’s R = 0.81) and 2-methyl-2-hydroxybutanoic acid (Spearman’s R = 0.62) were linked to increases in *Mobiluncus* spp. Putrescine was also linked to *Prevotella* spp. (Spearman’s R = 0.69), *Anaeroglobus* spp. (Spearman’s R = 0.61) and *Porphyromonas* spp. (Spearman's R = 0.60). Cadaverine (Spearman's R = 0.7) and putrescine (Spearman's R = 0.73) were both linked to increases in *Dialister* spp., and cadaverine was also linked to increases in *Streptococcus* spp. (Spearman's R = 0.84) and *Mycoplasma* (Spearman's R = 0.66). Diethylene glycol was linked to multiple genera, including *Stenotrophomonas* spp. (Spearman's R = 0.85), *Janthinobacterium* spp. (Spearman's R = 0.85), *Morganella* spp. (Spearman's R = 0.85), *Comamonas* spp. (Spearman's R = 0.84), *Delftia* spp. (Spearman’s R = 0.84), *Pseudomonas* spp. (Spearman’s R = 0.74), *Gardnerella* spp. (Spearman’s R = 0.67) and *Acinetobacter* spp. (Spearman’s R = 0.61).

In terms of demographic parameters, marriage formed a direct negative correlation with the Amsel-determined BV state (Spearman’s R = −0.48). However, most of the subjects' demographics and behaviors were many steps removed from either Amsel-determined BV type or a high Nugent score within the resulting positive association network (Table 5).

**Table 5:** Network path lengths to BV.

Feature	Shortest path			
	Amsel-defined BV-positive		High Nugent score	
	All	Positive only	All	Positive only
Metadata				
Married	1	n/a	2	n/a
Douching^1^	2	3	2	n/a
Condom use	9	n/a	3	n/a
Tampon use	4	n/a	4	n/a
Bath frequency^2^	n/a	n/a	n/a	n/a
Number of pregnancies	7	5	4	n/a
Last period^1^	4	5	3	n/a
Promiscuity^4^	3	5	3	n/a
New partner^4^	4	5	4	n/a
Recent intercourse^1^	3	5	3	n/a
Oral sex frequency^3^	5	6	4	n/a
Anal sex frequency^3^	3	6	4	n/a
Age	6	7	6	n/a
Vaginal sex frequency^2^	3	8	2	n/a

Measured as ^1^Days since ^2^Times weekly ^3^Times monthly ^4^In past 6 months.

## Discussion

Spiegel and colleagues [18] previously compared VFA profiles between BV-positive and asymptomatic women, finding an increase in succinate, acetate, butyrate and propionate with a concomitant decrease in lactate occurring with disease state. Consistent with this, our metabolomic analyses revealed that the most distinguishing determinant among samples from women with a high Nugent score and those with a low-moderate Nugent score was a marked decrease in lactic acid. Although, the relative proportion of lactic acid was not found to be significantly different among women delineated by the Amsel criteria (p>0.05). We also observed an increase in acetate with SBVI and propionate with SBVII. However, we did not observe significant changes in succinate or butyrate for women with BV-positive Amsel criteria, or those with a high Nugent score (p>0.05).

One rationalization for these observations could be a reduction in lactic acid-producing lactobacilli along with increases in other microbes capable of producing acetate and propionate in the vaginal tracts of BV-positive women. Decreases in lactobacilli have been described previously in women with high Nugent scores [19], as have increases in *G. vaginalis* [19], which are known to produce acetate and succinate [20]. The relative proportions of lactobacilli and *Gardnerella* spp. are generally considered hallmarks of the Nugent criteria [6]. Consistent with this, the relative proportions of *Gardnerella* spp. and lactobacilli 16S rRNA reads were highly discriminatory among samples with high and low Nugent scores. *Gardnerella* spp. were significantly more abundant in samples from women with high Nugent scores. However, while the abundance of lactobacilli was clearly reduced in several samples with high Nugent scores, our results did not indicate a significant reduction in overall representation of lactobacilli consistent with Nugent classifications. This finding may have been confounded by the inability of our sequencing primers to detect certain lactobacilli, such as *L. iners*. However, the addition of qPCR enumerations of *L. iners* did not affect these findings. *Dialister* spp. were also observed to be significantly more abundant in samples with high Nugent scores relative to those with low or moderate scores. Human isolates of *Dialister* spp. are reported to produce propionate [21], [22], presumably through the decarboxylation of succinate [22], which may influence the relative abundances of these metabolites in Amsel-positive samples.

The relative abundances of 16S rRNA reads from lactobacilli, *Gardnerella* spp., or *Dialister* spp. were not significantly different among Amsel-positive or -negative samples, suggesting a limited relationship between the relative abundance of these bacterial genera and disease symptomology. 16S rRNA reads belonging to *Gardnerella* spp. were detected in 50% of Amsel-negative samples, which is consistent with historical reports, where *G. vaginalis* could be cultured from 58%–68% of healthy samples [14], [15]. Yet, 16S rRNA reads pertaining to *Gardnerella* spp. were more commonly detected in women distinguished by either a high Nugent score or by positive Amsel criteria. These observations may reflect recent findings that different strains of *G. vaginalis* have different metabolic or virulence potentials [16], [23], with only a subset of strains being important to the onset of BV or defining the BV state.

In contrast to our diagnostic capabilities using 16S rRNA gene profiles, we were able to clearly delineate Amsel-defined BV-positive samples from BV-negative samples using metabolite profiling. Our analysis revealed the presence of two distinct metabotypes that are delineated by significant differences in 48 discrete metabolites. It was unclear if these metabotypes were affected by microbial or host metabolisms, but we did observe negative correlations between metabotype I and Ravel's biotype IV vaginal microbiomes [9], as well as biotype IV microbial genera including *Aerococcus*, *Atopobium*, *Gardnerella*, *Peptostreptococcus* and *Veillonella*. Amsel-defined BV-positive samples formed distinct groups within each metabotype, which we denoted as SBVI and SBVII.

We observed considerable overlap between the significantly affected metabolites of women with a high Nugent score and those with SBVI, suggesting that Nugent criteria were highly diagnostic for this diseased state. Consistent with this, all SBVIs had a Nugent score≥7. In contrast, no overlap was observed between the vaginal metabolomes of women with a high Nugent score and those with SBVII. In these samples, more than half had a Nugent score<7.

Women defined as BV-negative based on Amsel criteria, but having a high Nugent score, were observed in both metabotypes I and II. Our networking approach suggested that a high Nugent score was only indirectly connected to Amsel criteria, via a negative connection to sedoheptulose, a common urinary metabolite [24].

The most determinate metabolite for both SBVI and SBVII was a marked reduction in 1,2 propanediol, a hydrogenated derivative of lactic acid. This reduction reaction is carried out by some lactobacilli under pH conditions <5.8 [25]. Therefore, it is unclear whether 1,2-propanediol, and by extension the subset of lactobacilli capable of reducing lactic acid, is critical to vaginal health or if this merely reflects an elevated pH, as is characteristic of a BV state. The reduction of lactic acid also typically results in acetic acid at a 1∶1 ratio with 1,2-propanediol [26]. In agreement with this, we observed a marked reduction in acetic acid, but only in SBVI, which may reflect additional metabolic pathways that utilize acetic acid in the vaginal microbiomes of metabotype II women.

The second most determinate metabolite for each symptomatic BV type was 1-acetoxy-2-propanol, which can be resolved from 1,2-propanediol in the presence of lipase activity [26]. Many of the metabolites significantly affected in samples from women with a high Nugent score or displaying symptoms of BV types were fatty acids, including both SCFA and those with longer chain moieties. These and other metabolites found to be reduced in SBVI and SBVII (such as ethanolamine, glycerol, serine, phosphate) are related to glycerophospholipid metabolism and are highly suggestive of either reduced glycerophosphodiester phosphodiesterase-activity [27] or rapid utilization of these and other host cell membrane degradation bi-products (such as cholesterol; 2-aminoethylphosphate which were also significantly decreased) in the symptomatic BV state. Consistent with the latter hypothesis, increases in the biomembrane lipid anchor, tetradecanoic acid, an essential structural component of host cell membranes and their derivatives, was observed in the vaginal metabolomes of SBVI patients (Fig. 5). We also noticed significant differences in amino acids characteristic of integral membrane proteins. Amino acids with aliphatic side-chains, predominant in the transmembrane regions spanning the middle of the phospholipid bilayer (leucine, isoleucine, alanine and valine) were all depleted in metabolome samples from women with SBVI and/or those with a high Nugent score. While the amino acids tyrosine and tryptophan, which are typically located at the polar/non-polar interface of transmembrane protein regions were depleted in samples from women with high Nugent scores. The finding that these observations overlap with Nugent scores implies an important role for *G. vaginalis* and other Nugent-defined morphotypes in the reduction of cell wall integrity. In accord with this, the genomes of two *G. vaginalis* strains isolated from women with symptomatic BV harbor mucinolytic enzymes [16].

We tested the interrelationships among microbial genera, metabolites, host behaviors, disease symptoms, Nugent score, and clinician-determined disease state using a systems-based networking approach. Although the nature of these analyses precludes conclusive evidence, they potentiate significant new insight into the underlying mechanisms driving the disease process. Features uncovered by systems-biology approaches can then be explored using more conclusive analyses.

Our networking approach indicated connections between odor and the biogenic amines putrescine and cadaverine. Putrescine and cadaverine are both formed in fish and shellfish by bacteria during decomposition [28] and have been strongly correlated with the characteristic odor exhibited by spoiled fish [29], [30]. These matched the profiles of the characteristic odor of BV, described as having an amine or “fishy” smell [7]. Coordinated with increases in these metabolites, we observed decreases in volatile compounds reported to exhibit more pleasant odors, such as 2(5H)-furanone [31] and 2-ethyl-4-methyl-1,3-dioxolane [32]. Putrescine and cadaverine were not universally increased in SBVII metabolome profiles; however, odor was only clinically ascribed to one of these subjects, compared to all SBVI subjects. In this SBVII individual, levels of putrescine and cadaverine were higher than in any asymptomatic individual. The lack of correlation between odor and SBVII was an interesting observation and may partially explain the linkage of douching to this group. Two recent papers have described the ability of douching to reduce or eliminate vaginal odor [33], [34]. Douching was not inversely correlated with either putrescine or cadaverine; however, such a correlation would only be expected if these metabolites were found more universally in the absence of douching (i.e., if not douching would equally lead to an increase in these metabolites). Although douching had a significant affect on the compositions of the microbial community and the metabolome, it did not alone explain the delineation between metabotypes I and II.

We observed links between 2-methyl-2-hydroxybutanoic acid and discharge, as well as diethylene glycol and pain. The nature of these linkages is less clear, but it is worth noting that interpretation of metabolomic data can be difficult. Being only privy to metabolites above an appreciable level (typically metabolic end-products or those produced faster than they are utilized) means pathway intermediates, and consequently the pathways leading to these metabolites are not "observed". This makes a strong case for combining metabolomics approaches with metagenomics and metatranscriptomics.

We identified links between four metabolites that appeared important to symptomology (putrescine, cadaverine, 2-methyl-2-hydroxybutanoic acid, and diethylene glycol) and several microbial genera. For example, *Dialister* spp., detected in all SBVI samples, were linked to increased levels of both putrescine and cadaverine and *Gardnerella* spp. were linked to increased diethelene glycol levels, while *Mobiluncus* spp. were linked to increases in 2-methyl-2-hydroxybutanoic acid, supporting roles for these bacteria in defining the BV state.

With the exception of douching, most factors relating to subject behavior were many steps removed from BV-positive Amsel criteria or from a high Nugent score within the resulting positive association network (Table 5). We therefore hypothesize that, while personal habits and sexual practices may be influential, or even be essential precursors, additional factors contribute to the onset of BV. Marriage had a strong negative correlation with Amsel-determined BV-positive women, but we did not observe similar associations with promiscuity or sexual frequency, suggesting changes in these behaviors did not account for this observation. Many speculations could be posited for this, but we hypothesize it relates to findings that couples share more similar microbial types [35], such that intercourse between stable sexual partners results in less perturbation of the vaginal microbiome.

**Figure 8:**
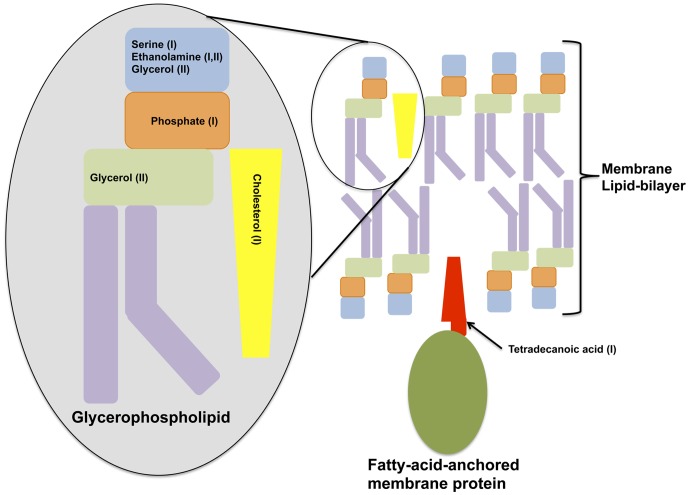
Cell Membrane Degradation. Shown is a diagram depicting classical components of a cell membrane that is depleted in either of the metabolome profiles of the two symptomatic BV types.

Overall we have shown clear delineations between the vaginal metabolomic profiles of women displaying BV-positive Amsel criteria and those from asymptomatic women. Our results hint at the possibility of two different paths to the symptomatic BV state, with each sharing a common disruption of host epithelial cell integrity, but differing in some symptoms, implicative microbial taxa and their detectability by the Nugent method (Fig. 8). Based on these observations and an accumulation of evidence over the years, it is becoming increasingly clear that the break down of a typically lactobacilli-dominated vaginal microbiome only increases susceptibility to BV. The onset of disease symptoms requires the presence or absence of certain metabolites that may be positively or negatively affected by certain microbial metabolisms. Furthermore, symptoms of BV (discharge, odor, itching and pain) may not have a common origin, but instead may each be related to separate metabolic processes elicited by the presence of *Dialister* spp., *Gardnerella* spp., *Mobiluncus* spp., or other bacteria. Findings that horizontal gene transfer is extensive within the vaginal microbiome [16] and that such genetic exchange has led to dramatic disparities in the virulence potentials of *Gardnerella vaginalis* strains highlight the murky relationship between BV and specific microbial taxa. Metabolomic profiling may offer a fast and cost-effective alternative to other methods enabling definitive diagnoses.

## Methods

### Ethics statement

The Institutional Review Boards of the University of Illinois at Urbana-Champaign (#05079, obtained 10-31-11) and the Carle Foundation Hospital (#89689-4, obtained 06-17-11) approved this study. Informed written consent was obtained from all study participants prior to sample collection.

### Sample collection

Ectocervical lavage (LA) samples were collected using a 15-ml sterile saline solution (Health Care Logistics, Circleville, Ohio) from 36 individuals, as previously described [13]. Patients were evaluated for BV via the Amsel criteria (vaginal discharge, pH>4.5, odor, clue cells; [17] by a trained clinician with >20 years experience prior to sample collection. Samples were frozen immediately upon collection and stored at −80°C for up to three years until used. Vaginal smears were also obtained for each subject at the time of clinical examination by rolling a swab across and along the length of the vaginal wall and then onto a glass slide, which was stored at −80°C until Gram stained for independent evaluation by a second trained professional, also with >20 years experience, according to the Nugent criteria [6]. A Nugent score of 7–10 was classified as having a high Nugent score and is often described as having a positive bacterial vaginosis (BV) state, irrespective of disease symptoms.

### 16S rRNA gene sequencing and analysis

Genomic DNA was isolated from 0.5-ml aliquots of each collected lavage sample. To each aliquot, 125 µl of 0.5 M Na.EDTA (pH 8), containing 75 mg/ml of lysozyme was added and samples were lysed at 37°C in for 30 min. To each sample, 70 µl of sodium-dodecyl-sulfate was added and protein was degraded by incubation at 55°C in the presence of proteinase K (0.05 mg/ml, final concentration) for 30 minutes. Further lysis was facilitated by three cycles of rapid freeze-thaw, performed by immersion in a dry-ice/ethanol slurry until frozen, followed by heating to 37°C. Protein was precipitated by the addition of 70 µl of NaCl and a 30-minute incubation on ice. Precipitated protein was removed by centrifugation and residual organic matter was removed by phenol:chloroform washes, followed by ethanol precipitation to remove residual salts.

The V1-V3 region of the 16S ribosomal RNA gene was amplified from the resulting DNA by polymerase chain reaction (25 cycles of 94°C (30 s), 48°C (30 s), 72°C (2 min)), using primers 27f-YM (CGTATCGCCTCCCTCGCGCCATCAG-AGAGTTTGATYMTGGCTCAG; and 534r (CTATGCGCCTTGCCAGCCCGCTCAG-(MID tag 1–50)-ATTACCGCGGCTGCTGGCA). The amplicons were pyrosequenced using 454 FLX-Titanium technology at the J. Craig Venter Institute (Rockville, MD). The resulting sequences were quality trimmed using the FastX toolkit (available: http://hannonlab.cshl.edu/fastx_toolkit/). Remaining sequences shorter than 250 nucleotides, with homopolymers longer than 6 nucleotides, or containing ambiguous base calls were removed. Sequences were aligned against the silva database [36]. Potentially chimeric sequences were detected using UCHIME [37] (<6% sequences detected as chimeric) and removed. The remaining reads were pre-clustered as previously described [38] and then clustered using ModalClust (https://bitbucket.org/msipos/modalclust). OTUs were defined as sharing ≥97% sequence complete-linkage identity with the most abundant sequence forming the OTU seed. OTUs detected in less than three samples and fewer than three times were removed as possible artifacts.

*Lactobacillus iners* was detected by PCR using primers 453F (ACAGGGGTAGTAACTGACCTTTG) and 1022R (ATCTAATCTCTTAGACTGGCTATG) [39] and conditions 95C for 10 minutes, followed by 30 cycles of 95C for 30 seconds, 65C for 30 seconds, and 72C for 30 seconds. A final extension was performed at 72C for 7 minutes. Samples positive for *L. iners* were then enumerated by quantitative PCR using the same primers with conditions 50C for 2 minutes, 95C for 10 minutes, followed by 40 cycles of 95C for 30 seconds, 65C for 30 seconds, and 72C for 30 seconds. Upon completion, a melting curve analysis was performed to ensure purity of the amplification product. All products showed the same overlapping peak indicating the specificity of the primers.

The raw sequences were deposited in the Sequence Read Archive (http://www.ncbi.nlm.nih.gov/sra) with accession numbers SRR639185.

### Metabolite analysis

Two 1-ml fractions of the lavage samples were taken from each subject and dried. One fraction was derivatized, according to [40] with minor modifications: 90 minutes at 500°C with 80 μl of methoxyamine hydrochloride in pyridine (20 mg/ml), followed by a 60-minute treatment at 500°C with 80 μl mass spec grade trifluoroacetic acid. A 5-μl aliquot of an internal standard (C31 fatty acid) was added to each sample, in the derivatized samples this occurred prior to trimethylsilylation. Sample volumes of 1 mL were injected with a split ratio of 7∶1 into a GC-MS system, consisting of an Agilent 7890A (Agilent Inc, Palo Alto, CA, USA) gas chromatograph, an Agilent 5975C mass selective detector and an Agilent 7683B autosampler. Gas chromatography was performed on 60-m HP-5MS columns with 0.25 mm inner diameter and 0.25 mm film thickness (Agilent Inc, Palo Alto, CA, USA) and with an injection temperature of 2500°C, the interface set to 2500°C, and the ion source adjusted to 2300°C. The helium carrier gas was set at a constant flow rate of 1.5 ml min^−1^. The temperature program of 5-min isothermal heating at 700°C, followed by an oven temperature increase of 50°C min^−1^ to 3100°C and a final 20 min at 3100°C. The mass spectrometer was operated in positive electron impact mode (EI) at 69.9 eV ionization energy in m/z 30–800 scan range. The spectra of all chromatogram peaks were then compared with electron impact mass spectrum libraries NIST08 (NIST, MD, USA), WILEY08 (Palisade Corporation, NY, USA), and to a custom library of the University of Illinois Carver Metabolomics Center. To allow direct comparisons between samples, all data was normalized to the internal standard in each chromatogram. The chromatograms and mass spectra were evaluated using the MSD ChemStation (Agilent, Palo Alto, CA, USA) and AMDIS (NIST, Gaithersburg, MD, USA). The retention time and mass spectra were implemented within the AMDIS method formats. Metabolites were determined to be significantly enriched in one or other state if after normalization, their median concentration in that state was >3-fold increased over the alternative state and they achieved a *p*-value of p<0.05.

### Data analyses

Taxonomic profiles from phyla to genus were generated for all reads using the RDPclassifier v2.4 [41], with a cutoff of 0.7. Species identities were determined using speciateIT (as described in [9]) and these designations were used to evaluate sample biotypes (I–V), as defined by [9]. *L. iners* qPCR results were added to speciateIT results based on their proportion of the total bacteria present in the vagina using prior enumerations of the total number of bacteria present in the human vagina (∼4×10^8^) [42]. Heatmaps were produced using the gplots package in R (Available: http://cran.r-project.org/web/packages/gplots/index.html), and dendrograms were constructed from Euclidean dissimilarity distances.

The relationships among the samples were compared using Bray-Curtis dissimilarity statistics, following normalization of the data to their total read depth (i.e., the proportional representation of each taxonomic group, or metabolite) and transformation of this data by square-root to reduce the influence of higher abundant over less abundant features. nMDS plots were constructed using this data and visualized in Primer (Primer-E 2007).

### Network analysis

For parametric analysis of patient metadata on hygiene and sexual practices with data relating to taxonomic abundances, including *L. iners* qPCR-determined abundances, and metabolite abundances, pairwise Pearson's product moment correlation coefficients were determined. For non-parametric patient metadata, including patients demographics and symptoms, pairwise Spearman's correlation coefficients were determined. Pearson's and Spearman's correlations were obtained using the ‘cor’ function in R. A set of 1000 random variables, each with 36 random values between 0 and 100 were added to the dataset prior to forming correlation matrixes. The correlations formed between these random variables and our dataset were used to evaluate the appropriate correlation thresholds (Fig. S3 and Fig. S4). Correlations >0.6 or <−0.4 were determined to be significantly discriminative and were mapped into a network by using cytoscape [43], after transformation into the appropriate format using custom perl scripts. Distances between patient variables were determined using Shortest path (Available at: http://www.cgl.ucsf.edu/Research/cytoscape/shortestPath/index.html).

## Supporting Information

Figure S1 ***Lactobacillus iners*** **-specific PCR.** Gel image shows the resulting products from a *Lactobacillus iners*-specific PCR. Sample positions are shown above the loading lanes.
(PDF)

Figure S2 **Overall Network.** The overall network of Pearson's (between parametric data) and Spearman's (between non-parametric and either parametric or non-parametric data) correlations >0.6 (green) or <−0.4 (red) are shown as edges connecting patient metadata relating to demographics, hygiene and sexual behaviors and sexual practices, OTUs, microbial genera, metabolites and patient symptoms. Sub-networks of this network are shown in text and in Fig S4.
(TIFF)

Figure S3 **Determination of Positive Correlation Thresholds.** Plot shows the proportion of potential connections realized as the correlation threshold increases for our dataset and compared to a set of 1000 random variables, each with 36 random values between 0 and 100. Using this approach we determined that positive significance thresholds of ≥0.6 for either Pearson's (parametric) or Spearman's (non-parametric) correlation coefficients were most informative of significant positive correlations.
(PDF)

Figure S4 **Determination of Negative Correlation Thresholds.** Plot shows the proportion of potential connections realized as the correlation threshold increases for our dataset and compared to a set of 1000 random variables, each with 36 random values between 0 and 100. Using this approach we determined that negative correlations of ≤−0.4 for either Pearson's (parametric) or Spearman's (non-parametric) correlation coefficients were most informative of significant correlations.
(PDF)

Figure S5 **Relationships between Nugent score, Nugent-determined BV and BV Symptomology.** Sub-network showing the two paths from Nugent score and Nuget-determined BV (Nugent score≥7) and symptomatic BV as determined by Amsel criteria. Positive connections are shown as green edges, negative connections are shown as red edges.
(TIF)

Table S1 Microbial taxa and their relative 16S rRNA gene representation.
(XLSX)

Table S2 Metabolites and their relative concentrations.
(XLSX)

Table S3 Table S3 Network Connections.
(TXT)
