# Supplementary figures and images for: A Multi-Omic Systems-Based Approach Reveals Metabolic Markers of Bacterial Vaginosis and Insight into the Disease
Source: PLoS One. 2013 Feb 6;8(2):e56111. doi: 10.1371/journal.pone.0056111 (PMC3566083; doi:10.1371/journal.pone.0056111)

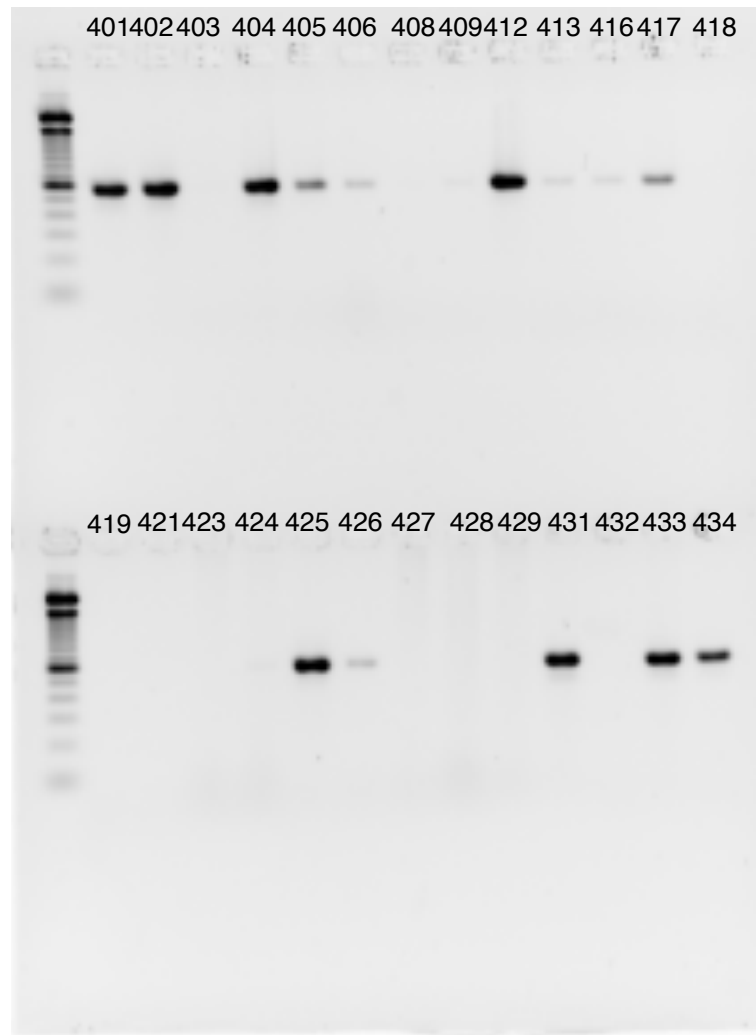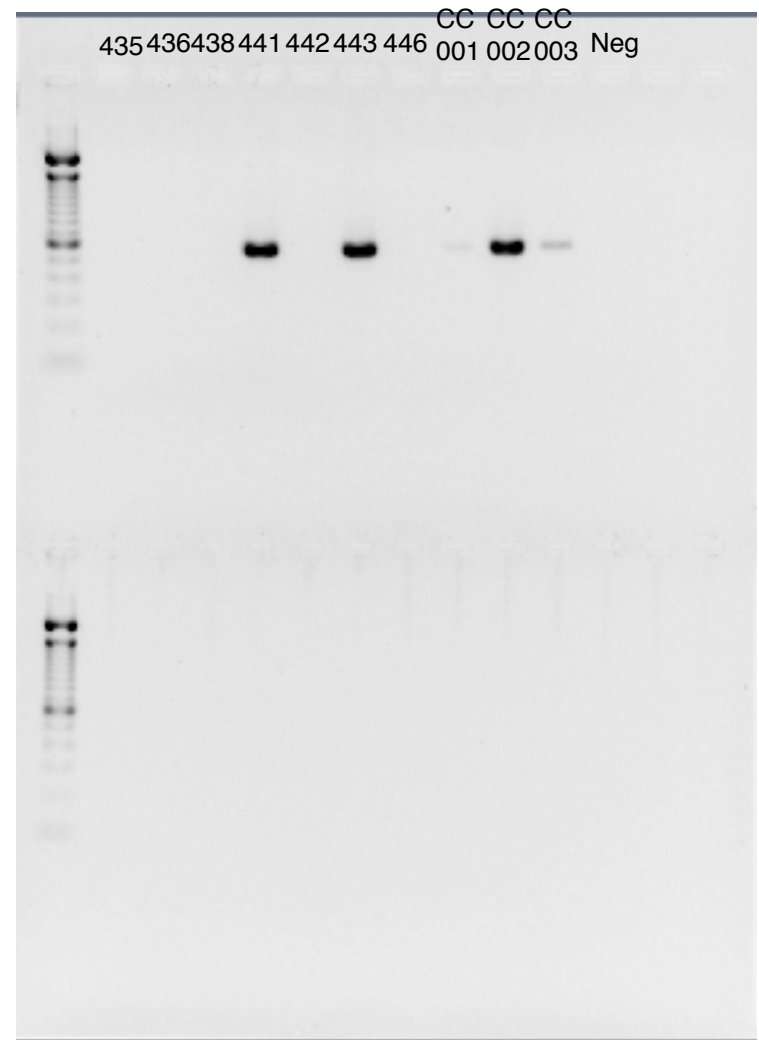

Supplement: Figure S1 — Lactobacillus iners -specific PCR. Gel image shows the resulting products from a Lactobacillus iners-specific PCR. Sample positions are shown above the loading lanes. (PDF) [file pone.0056111.s001.pdf]

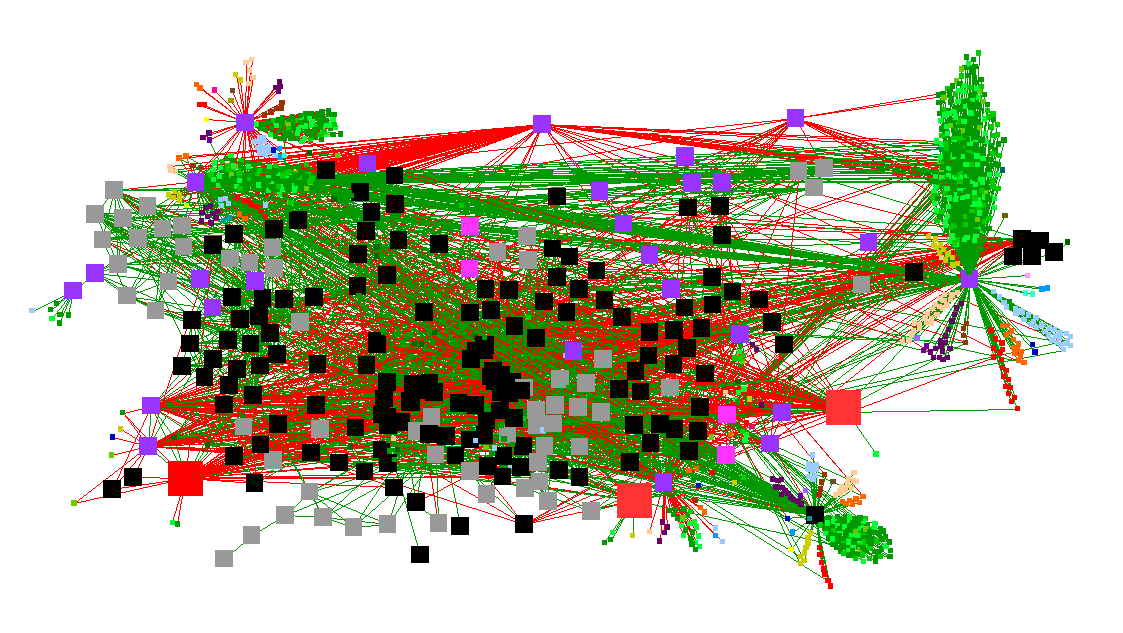

Supplement: Figure S2 — Overall Network. The overall network of Pearson's (between parametric data) and Spearman's (between non-parametric and either parametric or non-parametric data) correlations >0.6 (green) or <−0.4 (red) are shown as edges connecting patient metadata relating to demographics, hygiene and sexual behaviors and sexual practices, OTUs, microbial genera, metabolites and patient symptoms. Sub-networks of this network are shown in text and in Fig S4. (TIFF) [file pone.0056111.s002.tiff]

Proportion of potential connections realized

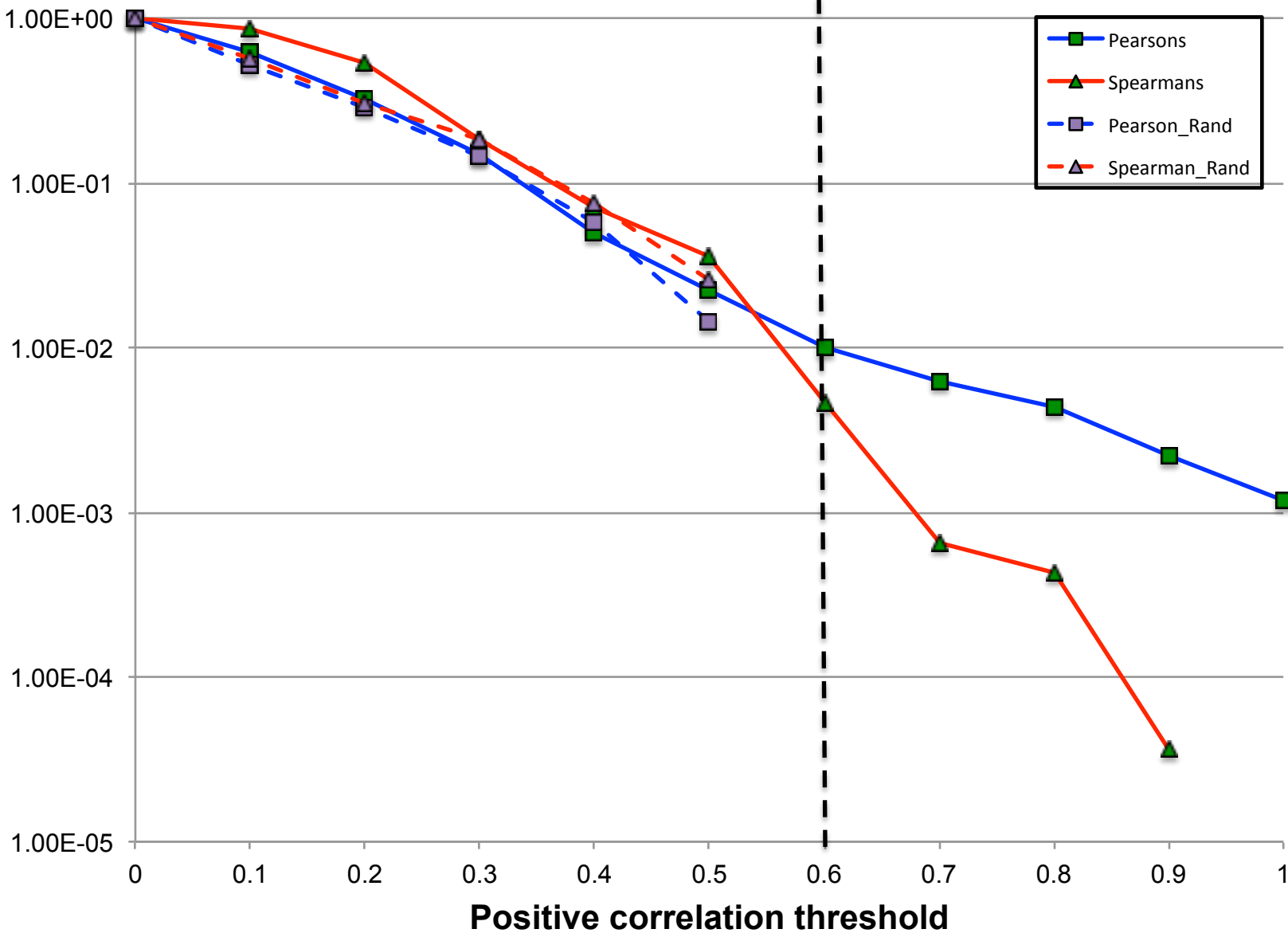

Supplement: Figure S3 — Determination of Positive Correlation Thresholds. Plot shows the proportion of potential connections realized as the correlation threshold increases for our dataset and compared to a set of 1000 random variables, each with 36 random values between 0 and 100. Using this approach we determined that positive significance thresholds of ≥0.6 for either Pearson's (parametric) or Spearman's (non-parametric) correlation coefficients were most informative of significant positive correlations. (PDF) [file pone.0056111.s003.pdf]

Proportion of potential connections realized

1.00E+00  
1.00E-01  
1.00E-02  
1.00E-03  
1.00E-04  
1.00E-05  
1.00E-06  
1.00E-07  
1.00E-08

0

-0.2

-0.4

-0.6

-0.8

-1

Negative Correlation Threshold

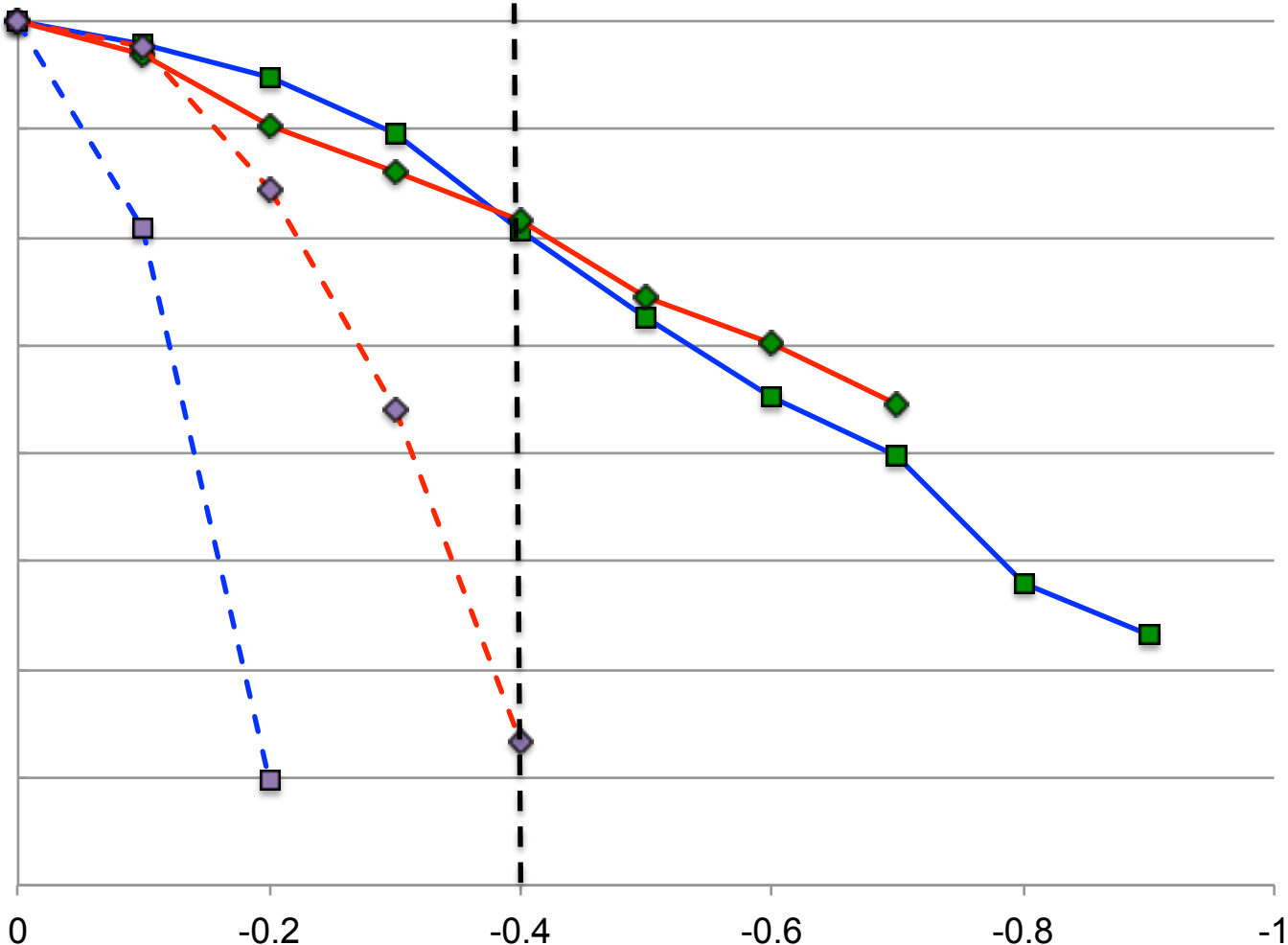

Supplement: Figure S4 — Determination of Negative Correlation Thresholds. Plot shows the proportion of potential connections realized as the correlation threshold increases for our dataset and compared to a set of 1000 random variables, each with 36 random values between 0 and 100. Using this approach we determined that negative correlations of ≤−0.4 for either Pearson's (parametric) or Spearman's (non-parametric) correlation coefficients were most informative of significant correlations. (PDF) [file pone.0056111.s004.pdf]

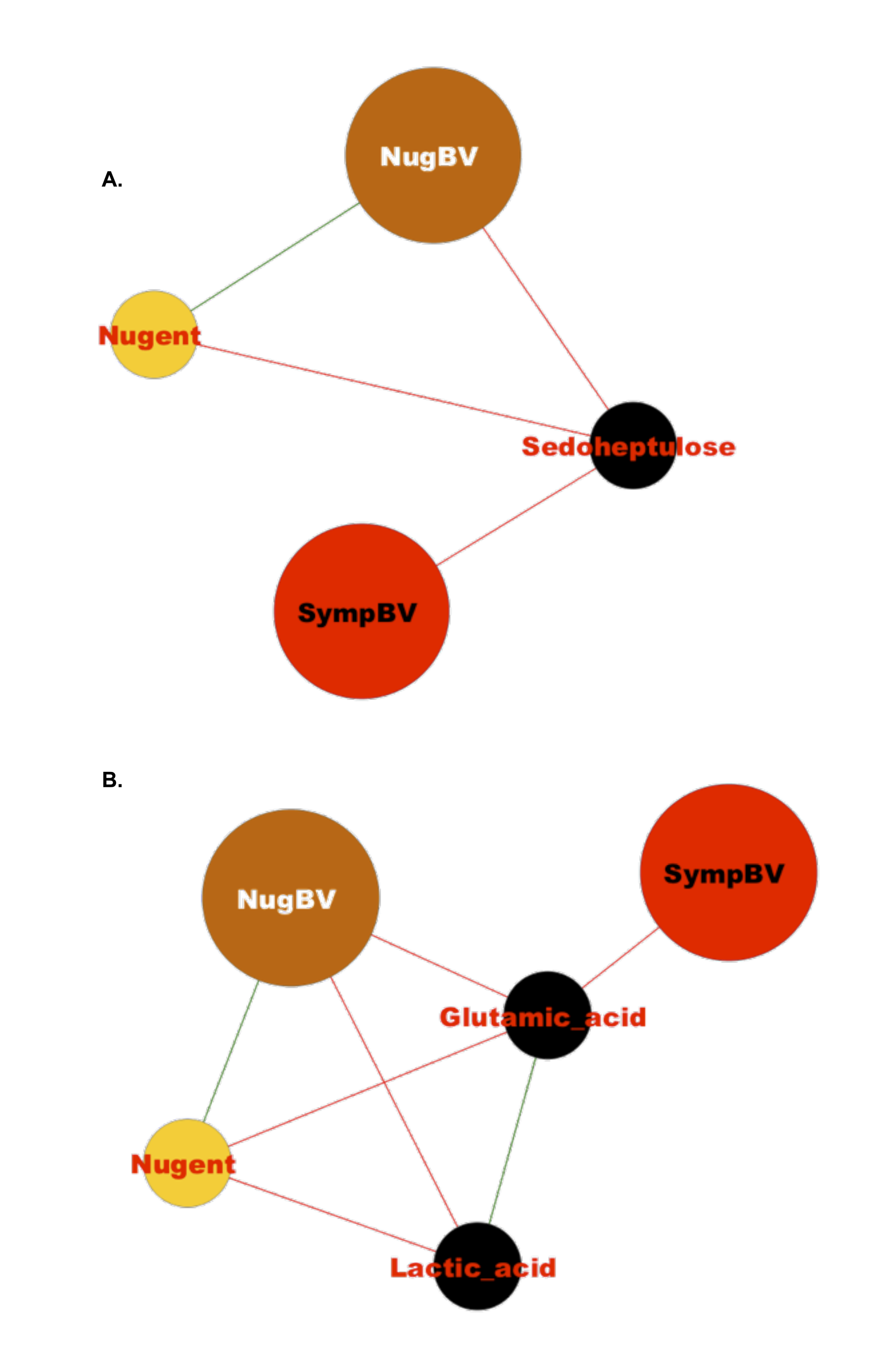

Supplement: Figure S5 — Relationships between Nugent score, Nugent-determined BV and BV Symptomology. Sub-network showing the two paths from Nugent score and Nuget-determined BV (Nugent score≥7) and symptomatic BV as determined by Amsel criteria. Positive connections are shown as green edges, negative connections are shown as red edges. (TIF) [file pone.0056111.s005.tif]
